# Hooping Cough

**Published:** 1855-08

**Authors:** Laurence Turnbull

**Affiliations:** Philad’a.


*Hooping Cough.* By LAURENCE TURNBULL, M.D. of Philad'a.

This disease has been to me one of much thought and considerable personal interest, from having had four of my children attacked with it in its most aggravated form. My attention was also particularly called to it during the months of May and June 1854, when the malady prevailed to a considerable extent in our city; cases of it have also continued to occur up to the present month, July, 1855.

In referring to the works of Hippocrates, (Sydenham Society's Edit. in two vols. London, 1849,) I find no description of hooping cough, neither is there any account of it in the Seven Books of Paulus Ægineta,* so that I would infer that the Greeks, Romans and Arabians were not acquainted with it as a distinct disease.

Dr. Willis was the first medical writer who accurately described hooping or chin cough, his work being published in 1682, (in two vols.)† It was not until the present century, however, that this disease was fully investigated and made known to the medical public, which was chiefly done by the labors of Rosen, Cullen, Schæfer, Hufeland, Mathaï, John, Authenrieth, Watt of Glasgow and Albers of Bremen. It is stated by Rosen, that it passed from the East Indies and Africa into Europe.

*First stage.*—The first stage of hooping cough has no distinct and prominent symptoms by which it can be distinguished from ordinary catarrh, or bronchitis, except, perhaps, a slight difference in the voice and cough which sounds louder and shriller; the expectoration is usually limpid, but in some instances I have noticed it opaque, yellow, and even greenish.

This period may last from five days to as many weeks. Lombard states that in an epidemic which occurred at Geneva, it lasted from one month to six weeks; when the hoop is going to occur, it is usually noticed in the second or third week, but I have had several cases where the cough was present without the hoop.

* The Seven Books of Paulus Ægineta, translated from the Greek, by Francis Adams, L. L. D., in three vols. London, printed for the Sydenham Society, 1844.

†" Tussis puerorum convulsiva, sue suffocativa et nostro idiomate *chin-cough* vulgo dicta." (Opera Omnia, Amst. 1682, vol. ii. p. 169.)

This absence of the hoop is often very unfortunate, for children may in this way propagate the disease, and cause whole families and even schools to be attacked. This fact was proven in the case of my son, aged seven years; he sat in school by the side of a little boy who had a cough, which was very sonorous and painful to listen to, but the anxiety of the teacher was much relieved by being informed that his physician did not consider it hooping cough; subsequently the youngest child of this family was attacked by the disease and hooped, and the boy was then kept from school, but too late to save the other members of his class, ten of whom took the disease, so that the school was nearly broken up; my son communicated it to his three sisters, who all suffered more severely from it than he did.

I therefore think that children should not be allowed to mix with their companions when suffering from a cough of this character, if the disease prevail in the locality.

*Second Stage.*—The second or spasmodic state of this malady, is easily known, by the peculiar sound and suffocating character of the cough. In this stage almost every organ is irritated, and it even produces discharges of blood from the nose and mouth. The expression of the countenance is most distressing. When this stage is at its height, the child seems to know by some inward sensation that the attack is coming on, and it either cries or lays hold of some object by which it can support itself until the paroxysm is over. The face and neck become swollen, and in some instances remain so, and the child, at the termination of a fit of coughing, either discharges some thick tenacious ropy mucus, or evacuates the entire contents of the stomach.

The least mental excitement, either of joy or sorrow, will induce an attack, and the number varies with the severity of the disease. The paroxysms last from one fourth to three fourths of a minute.

The average duration of hooping cough is from six to eight weeks if not checked, but in many instances it lasts as many months; second attacks are rare and yet they do occur. Hooping cough prevailed in this city with measles, in May and June, 1854, followed in October and November by chicken pox, and in January, 1855, by scarlet fever, with sporadic cases of catarrh and croup, showing a connection or relation one with another, so that the same causes may give rise during an epidemic to simple catarrh, croup, hooping cough, or even measles.

The complications of hooping cough I cannot enter into, but the chief of them are croup, bronchitis, pneumonia, pleurisy and diseases of the brain and cavity of the abdomen, which are to be recognized by their characteristic symptoms.

According to Billard, post-mortem examinations have not revealed anything uniform in this disease, except bronchial catarrh in its various stages.

Sydenham imputed the disease to a subtle and irritating vapor in the blood. Hufeland considers that the eighth pair of nerves is diseased, and is the cause of the double irritation of the bronchia and stomach. According to M. Guersent (Dict. de Méd.) hooping cough is a catarrhal affection, seated in the trachea and bronchi, consisting of a specific inflammation, accompanied with spasm of the trachea and glottis. Dr. Watt, of Glasgow, considers the disease to be inflammatory and seated in the bronchi. Albers, of Bremen, considers hooping cough to be an affection of the nerves of the thorax, with which bronchitis is frequently complicated. Læennec regards it as a variety of pulmonary catarrh, and from the convulsive character of the cough he calls it convulsive catarrh.

Dr. Webster (Med. and Phys. Jour.) is of the opinion that the symptoms, when closely viewed, suggest the impression that hooping cough depends upon inflammatory irritation of the brain or its membranes. This is the opinion held by Dr. Copland, and very many distinguised men of the present day, but to my mind it is not satisfactory. The hooping cough, in its first stage, is certainly of an inflammatory character, chiefly affecting the lining membrane of the air passages, but this is of a specific nature. In the second stage there is no evidence of inflammation indicated either by the pulse, skin, or any other organ, but there is a powerful irritation of the laryngeal constrictor, and bronchial muscles and nerves, producing a cough which occurs rapidly many times so that a single inspiration is followed by five or six successive expirations constituting paroxysms of coughing (tussis accessus), accompanied with redness of the face, watery eyes, headache, tinnitus aurium, fulness of the cervical veins, retching and sometimes vomiting.

By some writers it has been considered that this disease was produced by a peculiar miasma, acting chiefly on the nerves, and is also ascribed to the presence of minute insects in the air (Boehme—Linnæus,) or, according to Prof. J. K. Mitchell, its epidemic origin may be a peculiar fungus ; "the spores of these plants are not only numerous, minute, and indefinitely diffused, but, like the animal, all have the power of penetrating into and growing upon the most interior tissues of the human body," passing into the systems of those exposed to its influence by the respiratory organs or stomach, producing the irritation of the mucous membrane of the air passages. "Introduced into the body through the stomach, or by the skin or lungs, cryptogamous poisons are known to produce diseases of a febrile character, intermittent, remittent and continued—and even the disease of the mucous membrane termed aphthæ, arises from the presence of minute fungi."*

Dr. M. has not made this application of his doctrine to this particular disease, yet I do not see any good reason why it should not be so applied. According to Dr. Spengler, of Ellville, epidemic diseases depend on the presence or absence of ozone. He states that in the village of Roggendorf, in Mecklenburgh, towards the close of 1846, when slight catarrhal affections became prevalent, but slight traces of ozone were to be detected in the air. With the opening of the following year, however, these catarrhal affections assumed the severest forms of tracheal and bronchial disease, and hooping cough became common both among children and adults; then reagents detected a great increase of ozone in the atmosphere."†

*Prognosis.*

The prognosis in uncomplicated hooping cough is very favorable, and is unfavorable, only, in proportion to the dangerous nature of its complications and the age of the child; the best season for a favorable termination is spring or summer.

*The modes of Propagation.*—The disease may occur epidemically or sporadically, and it possesses infectious properties. It is propagated through a family from one to another ; they are not all apt to be attacked at the same time, and by removal to a dis-

* On the Cryptogamous Origin of Malaria and Epidemic Fevers. J. K. Mitchell, M. D. Philada. 1849.

† Lond. Med. Gaz. Henlie's Zeitschrift, vol. vii. p. 1.

tance a child may escape. Dr. Cullen believed that it disappeared in from four to six weeks, but this has not been proved by subsequent observation. Children who have suffered from this disease, should not be sent to school or play with their companions for at least two months.

*Treatment.*—There are but two classes of symptoms to be combatted in this disease when no complication exists. The inflammation must be reduced by depletion, expectorants and refrigerants. In the second class of symptoms, the chief object is to diminish the abundant secretion and allay the great irritability of the laryngeal constrictor, and bronchial muscles and nerves.

The means to accomplish this, in my hands, has been the abstraction of blood; the application of a few cups or leeches to the nape of the neck or under the clavicle, with counter irritation, by means of sinapisms and blisters, which will soon allay the congestion of the brain or lungs. To diminish the febrile action small doses of tartar emetic, combined with Dover's powder or prepared chalk, with the free use of syrup of ipecacuanha as an emetic may be given; these will lessen the bronchial inflammation, and remedy the often disordered state of the stomach and bowels.

During the whole stage of the disease, demulcent drinks should be freely administered, such as flax seed tea, barley or rice water. When fully satisfied that the inflammation has been subdued, indicated by a slower pulse, less heat of skin and no active congestion of the brain or lungs, I have then followed the treatment with belladonna, and my success with this remedy has been most gratifying. Before administering it I tried, in vain, the free use of cochineal in combination with alkalies, assafœtida, opium, alum, hydrocyanic acid, &c. In every instance in which the system was fully brought under the influence of the belladonna, indicated by dilatation of the pupil with confused vision and reddened skin, I was enabled to check the annoying cough and hoop of thirteen children during the months of May and June, 1854, and seven cases since that time, making twenty cases in all, eight males and twenty females; the youngest was nine months and the eldest ten years.

The following was the method followed: The system being prepared by reducing the inflammation by the means before spoken of, obtain, if possible, English extract of belladonna, fresh and good; let the extract be triturated with water or simple syrup; if it is to be kept for some time, add a small quantity of alcohol. The dose for a child three months old is the sixteenth of a grain every three hours, to a child one year one eighth of a grain, and so to other ages in proportion.

Inform the parent or nurse of the change it will produce upon the eye, also that it may redden the skin. When full dilatation of the pupil is brought about, the medicine is to be intermitted until it has gone off again; the belladonna is to be administered in slightly increasing doses, so as to keep the child under its influence for several days or until the paroxysms are checked, which will usually occur towards the sixth or eighth day of the second stage.

In the twenty cases cured by the use of the belladonna the cough and hoop returned in a few cases on exposure to cold, or in disagreeable, windy weather; but by combining the extract with syrup of ipecacuanha a few doses soon checked the cough and hoop; in only one case out of this number was it complicated with inflammation of the lungs, and this case recovered.

The average duration of my twenty cases was ten days, after the hoop had commenced when the case was free from complications, which shows the great advantage of this treatment. The ordinary duration of the disease, when treated in the usual manner, is from 1½ to 3½ months; even by prussic acid, or the application of nitrate of silver, the average given is from two to three weeks. It is stated by Dr. Gibb that, with the use of nitric acid, the average duration was only six or seven days. Several physicians who have used this remedy, however, do not find such favorable results from its use.

I have added to my communication some extracts from the experience of a few distinguished medical men on the use of this important agent, belladonna.

This remedy was used in hooping cough about the year 1783 by Dr. Buckhaave, of Copenhagen, who gave the powdered root in doses of two grains, morning and evening, to a child of five or six years of age. The cure, it is stated, was generally accomplished in from seven to fourteen days.*

*Dr. Duncan's Commentaries for 1793, and Dr. Gibb on Pertussis, p. 282, 1854.

Dr. Miquel, (of Neuerhaus,) says the belladonna is a remedy upon which he can always depend in this disease. In the course of many epidemics which he has observed during fifteen years, he has constantly cured the cough in eight days.*

Dr. Samuel Jackson, of this city, late of Northumberland, who although he was not the first to employ the belladonna, yet by his valuable publication in 1834 brought its virtues prominently before the medical public, has continued its use for twenty years, and his confidence in its powers to arrest the paroxysm and cure the second stage of hooping cough in the great majority of cases is undiminished.

Dr. Hiram Corson, formerly President of the Medical Society of the State of Pennsylvania, a distinguished practitioner of Montgomery county, Pa., in a paper on the efficacy of belladonna as a remedy in Pertussis, published in the Amer. Jour. of Med. Science, for Oct. 1852, makes the following observations: "My experience in pertussis had satisfied me that of all the remedies in common use, those recommended by writers upon diseases of children are almost useless. Children affected in the winter continued to cough and strangle and suffer for many weeks with scarcely a perceptible amendment. It was painful to visit and mortifying to prescribe for those afflicted with this malady."

He commenced the use of belladonna in four cases, and in one week they were all well. His method of using it was to begin with the sixteenth of a grain to children under one year every two hours, and increasing a little every day until full dilatation of the pupil occurred, the skin became flushed and vision confused; this he accomplished by dissolving eight grains of the extract in an ounce of water, nine drops of which contained the eighth of a grain.

In an epidemic in 1840, he used the belladonna in hundreds of cases with great relief in nearly all. By giving it in small doses at first, the fears of the patients were allayed. In 1847–8 he also prescribed it in numerous cases with much success. He concludes his paper in these words: "During the last seventeen years, I have given the extract of belladonna to hundreds of patients from two months to fifty years of age, and am firmly

*Vol. vii. Amer. Jour. Med. Sciences, p. 524, from Archives Generales, August, 1830.

convinced that it has a greater control over hooping cough than any other remedy in common use. That while, in a few cases, the system did not seem susceptible to its action in the doses I have prescribed, yet in nearly all the disease yielded quickly. It is a safe and efficient remedy for pertussis in children of any age.

Dr. Eberle, in his Treatise on the Diseases of Children, second edition, remarks "that the belladonna has been highly celebrated, and is without doubt, by far, the best article of the kind we possess. My own experience leads me to testify confidently on this point. I have prescribed it within the last six years, (1834,) in perhaps twenty cases, and in the majority of them with evident advantage." Professor Borda, he remarks, was the first he believed who used it as a remedy, and his belief in its efficacy is almost unlimited.

Hufeland and Alibert are almost equally decided in their praise of the virtues of this article.

The mortality from this disease in our city in 1850 was 114, 1852, 168; and for 1853, was 64. In 1853, in the district of Richmond it occurred as an epidemic. In severe cases, Dr. Janvier used the belladonna with the best results. "It mitigates the paroxysms better than any other sedative."*

Dr. Condie remarks in his work on diseases of children, that the narcotic from which the greatest amount of benefit is to be anticipated in this disease, is unquestionably the belladonna; it has been very extensively employed, and the evidence in its favor is strong and conclusive, (by Kahleiss, Janin, Hufeland, Widemann, Raisin, Guibert, Alibert, Shäfer, Laennec, Müller, Blache, Maunsell and Lombard.)

He further remarks that he had given the belladonna a very fair trial, and has, in many cases, been pleased with the prompt and decided relief produced by it, while in other instances it appeared to exert no influence whatever."

I think that this last remark may be often accounted for by the bad character of the belladonna, which is even found in some of the drug stores of this city, for it is an uncertain preparation unless when procured by evaporation in vacuo, for some samples from some of the Parisian shops were found by Orfila to be quite inert.

*Report Phila. County Med. Society, for 1853.

Dr. Williams, of London, has used belladonna with great advantage in his practice. He gives it in quarter-grain doses to a child of two years, increasing the dose to double that quantity or more, where it fails to relieve. He remarks that these doses, in general, cause some dilatation of the pupil, and conceives that the remedial agency of the drug depends on the same power to diminish irritability of the bronchial and laryngeal muscles which is here evinced with regard to the iris."*

Dr. G. A. Rees has found belladonna one of the most efficacious remedies in Pertussis.†

Dr. Waller cured two cases with the twelfth of a grain of extract, three times a day; prussic acid and conium had failed in affording any permanent relief.‡

Aberle assigns the highest place among narcotics to be belladonna in hooping cough.

Dr. Churchill says that this is perhaps the most influential narcotic and sedative we possess (in pertussis); it has been very extensively employed and the evidence in its favor is very strong.§

Belladonna has been eminently useful in the epidemics of hooping cough which M. Debreyne has observed, but recourse should not be had to it until the inflammatory element has been overcome by leeches, emetics, &c.

Dr. A. T. Thompson says, I have ordered the extract of belladonna in doses of one-eighth of a grain to a child of eight years, and gradually increased the dose to a quarter of a grain. Its power over the cough is extraordinary.||

I might bring forward the testimony of many other writers, and a mass of evidence from medical practitioners, to establish still more firmly the fact of the efficacy of belladonna in this peculiar malady, but it will not, I trust, be necessary.

I will now endeavor to give an epitome of the experience of the best writers in the treatment of hooping cough by means of other agents.

The first of these which I will notice is *alum*, which has been

*Gibb on hooping cough, p. 284, from Medical Gazette, Feb. 1838.

†Diseases of Children, 2d edition, 1844.

‡Lancet, vol. 1, 1845, p. 137.

§Elements of Materia Medica.

||London Jour. of Med., April, 1850.

very highly recommended by Dr. Golding Bird; it has been employed with success by Dr. John F. Meigs, of this city, who speaks of it as follows, in his useful work on the diseases of children: "From reading Dr. Bird's remarks on alum, and prompted by my knowledge of its admirable qualities in the treatment of croup, I was led to make trial of it in the disease under consideration, and I believe I may say that it has exerted a more decided influence in moderating the violence of the disorder than any that I have ever made use of. I have administered it in fifteen cases, beginning in the course of the second stage. In all it was beneficial, and in some the effects were strikingly useful, the improvement being more rapid than I had ever seen to result from other remedies." Dr. Bird gives from two to six grains every four hours. His formula is as follows: ℞. Aluminis, gr. xxiv; Ext. Conii,* gr. xii; Syrup Rhœados, ʒii; Aquæ Aneth. f.℥iij. ♏. Give a medium-sized spoonful every six hours. Dr. Meigs gives it in smaller doses, and without the Ext. Conii. To children under one year, half a grain to a grain three or four times a day; and to those over that age, two grains every six hours.

Dr. Crossly Hall, an English physician, employs the *alum* in powder, prescribed in a little water eight times a day, and he considers it a very useful remedy.

Dr. Davis highly extols the efficacy of alum in pertussis. In the late edition of Underwood's Treatise, edited by him, he says, "After a long trial, I am disposed to attach more importance to *alum*, as a remedy in hooping cough, than to any other form of tonic or antispasmodic. I have often been surprised at the speed with which it arrests the severe spasmodic fits of coughing; it seems equally applicable to all ages, and almost to all conditions of the patient. The dose for an infant is two grains three times a day; and to older children four, five and up to ten grains may be given mixed with syrup and water."

I have employed *alum* both in the case of my patients and my own children, and gave it freely; it moderated the intensity of the disease, but it did not in my hands make a cure, so that,

*This is considered by Dr. Butter as *the* remedy, namely conium, for hooping cough, and he eulogizes its use.

after its use, for ten days, I had to resort to the belladonna, which in a week completely checked the hoop.

Another agent which has been very highly lauded is the hydrocyanic acid, which is considered by Dr. Thompson, of London, to possess a "specific power" over the disease.

Dr. West, of London, author of a valuable treatise on the diseases of childhood, says, "that the acid sometimes exerts an almost magical influence on the cough, diminishing the frequency and severity of its paroxysms almost immediately, while, in other cases, it seems perfectly inert; and again in others, without at all diminishing the severity of the cough, it exerts its peculiar poisonous action on the system so as to render its discontinuance advisable."

He recommends it to be given by itself, diffused in a little distilled water, sweetened with simple syrup, and the dose he begins with is half a minim every six hours for a child nine months old. He has never but once, however, seen really alarming symptoms follow its use, though he has employed it in many hundred cases; still he remarks that although the severity of the cough may be relieved by the acid, it does not enable the practitioner to dispense with other remedies.

Dr. Hamilton Roe, in his treatise on hooping cough, gives to an infant three quarters of a minim of hydrocyanic acid, Scheele's strength, gradually increasing it to a minim, which is administered every four hours; for a child of three years of age one minim, gradually increasing, if necessary, to a minim and a half every four hours. Dr. Roe says he is convinced, from the result of all the trials he has made, that this drug will cure almost any case of simple hooping cough in a short time. Dr. Edwin Atlee first used it in 1824, and from that year until March, 1832, he says he has treated more than two hundred patients, and never failed to cure in from four to ten days.*

This medicine is highly recommended by Muhrbeck, Kahleiss, Volk, Heller, Granville, Lombard, &c. I have tried this acid, but it did not at all please me in its effects.

Another remedy which demands our notice is the precipitated subcarbonate of iron, (ferri sesquioxidium.) The following

*Amer. Jour. Med. Sci. vol. vii. and vol. x.

observations on its use are by Dr. H. C. Lombard, of Geneva, who, after praising the virtues of assafœtida, flowers of zinc, opium, prussic acid and belladonna, says, "I come now to my specific, or rather to the remedy advised by Dr. Steyman, as the best antispasmodic in hooping cough. Dr. Steyman had advised to give from four to ten grains of subcarbonate of iron in the twenty four hours, he gave as a rule to increase one grain for each year, so that a child six years old was to take six grains in a day, but from the beginning I found the dose quite inadequate, and I increased it to twenty-four and thirty-six grains in young children. I have given it either with water and syrup or mixed with a cough mixture. It has never produced any inconvenience. On the contrary, I have found that all the children treated after this method were much less weakened, and recovered faster than with all other remedies. The proofs of the advantageous effects of it have been so numerous that I can scarcely enter into the detail; however, I may give a few facts to corroborate my assertion. In a child four years old I gave the subcarbonate of iron, and the fits, which in the preceding week, had been 101 in number, were reduced to 66 in the following week. In a weak and debilitated boy, aged seven years, the powder of belladonna had proved quite useless, when I tried the powder of iron, so prompt was the effect that in a few days the boy was quite cured; the sister of this boy was also cured with great rapidity. The last case of hooping cough which I have treated lately was of four months duration, and every thing had proved useless, when I gave the iron powder, which in the space of a few days succeeded in making the cough less and less.

In fact, I think I may assert with security, that the subcarbonate of iron enjoys a remarkable property to make the fits less violent, to diminish the number, and after a certain number of days to cure entirely the hooping cough.

It enjoys, besides, the advantage of strengthening the little patients, and gives them force to resist a complaint which sometimes lasts some weeks, and generally leaves the patients weak, low and exhausted. In some of those who have taken it, I have often seen, during the first days, a temporary increase of the cough, but it always subsided after two or three days, and did not prevent the good effects of the medicine. The good effects obtained by the use of the iron powder are easily explained by its antiperiodic and antineuralgic properties, and it shows a posteriori how much the hooping cough resembles a true neuralgia, or, at all events, a true nervous disease."

I have not tried this remedy, and can, therefore, give no opinion of its efficacy; but should judge from its tonic and blood-restoring properties, that it would prove a useful agent in low anemic or debilitated cases.

Garlic is a remedy very highly recommended by Dr. Dewees; indeed, he states in his work on Diseases of Children, "that he has never employed any remedy of equal efficacy."

A child of six or seven years may begin by taking a third of a common sized clove, morning, noon and evening, in the absence of all febrile excitement, gradually increasing the dose.

Mr. Sutcliff combined the Peruvian bark with cantharides, and administered it with great success in hooping cough during twenty years.

The following is his formula:

℞. Tinct. Cort. Peruv. f. ℥vi.<br>

Elix. Paregor. ℥ss.<br>

Tinct. Canthar. ʒi. M.

Of this mixture, small doses were given three or four times a day, gradually increasing until a slight strangury was excited, then the dose was to be diminished.

When the active symptoms have subsided, Dr. Beatty, of Dublin, used the same remedy, and it is also recommended by Dr. Graves. The following is his formula:

℞. Tinct. Cinch. C. ℥v.<br>

" Lytta,<br>

" Camphora, *aa.* ℥ss.

M. S. A teaspoonful 3 times a day in flaxseed tea or barley water.

Professor Trousseau recommends the following solution of nitrate of silver in hooping cough: one-fifth of a grain in solution with simple syrup daily.

Cauterization by nitrate of silver has also been employed as a remedy in pertussis by Dr. Eben Watson, of Glasgow. The strength of the solution is gr. xv. to the ounce of water, applied every second day, by means of whalebone tipped with sponge, at first to the pharynx, and then to the glottis and larynx. The whole number of cases treated by M. Joubert and Dr. Watson, in 1854, were 167.

| | | | | |
|---|---|---|---|---|
| Cured in two weeks, | 96 | cases, | or 57.4 | per cent. |
| " in three or four weeks, | 61 | " | 36.5 | " |
| Resisted the treatment, | 9 | " | 5.3 | " |
| Died, | 1 | " | or nearly 0.6 | " |

To prevent the irritability of the stomach, he gives frequent small doses of heavy magnesia, combined with a few grains of the tris-nitrate of bismuth. He also employs the index finger or teaspoon to make the application to the throat of children.

Nitric Acid was first recommended by Dr. Arnoldi of Montreal as a remedy in pertussis with much success, and it has been adopted by Dr. Gibb, of London, late of Canada, who has published a work on hooping-cough in which he has given the opinion of ninety-three physicians in relation to its pathology, with its history, mortality, complications and causes. He has also entered into a consideration of forty-three remedies, viz., venesection, leeches, emetics, antimonials, external applications, change of air, warm bath, hydrocyanic acid, laurel water, belladonna, opium, hemlock, henbane, digitalis, tobacco, arsenic, silver, iron, zinc, lead, copper, cauterization by nitrate of silver, inhalations, coffee, Peruvian bark, quinine, hydrochloric acid, sulphuric acid, nitric acid, cochineal, alum, tannin, vegetable acids, alkalies, vaccination, cantharides, musk, assafœtida, meadow narcissus, cup moss, castor, nux vomica, and miscellaneous remedies. He remarks that the nitric acid has succeeded over and over again when other means have failed, and it is not such a hazardous remedy, when administered with ordinary precaution, as many described by him in his work. Dr. Arnoldi's method of prescribing the acid is as follows:

"To a tumbler full of very sweet water (almost syrup) add as much acid as will bring the water to the strength of pure lemon juice, when it is ready for use; an adult may consume this quantity in three or four hours, a child one year old may take a desert spoonful every hour."

"He has remarked that the efficacy depends on the amount taken, and that especially by the frequency of repetition, to save the teeth, he advises a solution of carbonate of soda, two drachms to eight ounces of water to be used as a gargle immediately after taking the acid."

"The object entertained by Dr. Arnoldi, in using this acid as a remedy, was to introduce the elements of the atmosphere into the blood by the process of gastric digestion, so as to enable the lungs to outstand the stage of temporary asphyxia. Whether the theory be correct or not, the result, he says, of his practice has been almost universally successful." He then goes on to give the outlines of twelve cases which were treated by Dr. A. with success. The doctor met with a few cases where the disease seemed to resist the action of the acid, owing, he remarks, to "spinal torpor at the track of the eighth pair and phrenic. In these the application of an ointment of the biniodide of mercury, so as to produce the specific eruption, and this reproduced a second and a third time, completely restored the efficacy of the acid."

"Dr. Gibb's own cases were sixty-four in number, which are reported as cured; he combines the acid with honey or syrup and compound tincture of cardamom, &c."

Chloroform has been employed by Dr. Fleetwood Churchill, as a specific remedy in hooping cough in four cases which he reports; in two of these the hoop ceased in two days; in the third case it required its use for three weeks; in his fourth case the patient had to resort to the use of Prussic acid to complete the cure.

"In the case of young children he drops thirty drops on the palm of the hand, the mother to hold this before the mouth and nose of the child sufficiently near to inhale it fully, but not so close as to exclude a portion of atmospheric air. The best time to begin is just as the patient feels the irritation in the chest increased to a cough.

Still he considers it more suitable for young persons of twelve or fourteen years old and upward. Two successful cases have come under my notice; the method was by placing a small portion of chloroform in a vial, and when feeling the inclination to cough, to inhale by removing the cork, the small bottle being carried in the pocket."

Before concluding my remarks upon the treatment of this disease, I must not neglect to state the great importance attached by some authorities to a change of air in the last stage, or the debility which results from it. Dr. Lombard remarks, that "in many cases which had baffled all attempts to stop the cough, a change of air has accomplished the cure. I have found it equally indifferent to go out of town or to come into town, provided there be a change ; and even in the short distance of half a mile, I have seen the good effects of this plan of treatment." Dr. West, of London, says, that change of air with the use of alum during the last stage, generally expedites the cure." According to Dr. Gregory, change of air after severe and protracted cases is the only thing that will give the patient a chance of recovery.

Billard states "that goat's milk, pure or diluted, a good nurse, a residence in the country, particularly in the spring and summer, will materially conduce to the recovery of infants at the breast."

But nothing can be more pernicious than the exposure of children suffering from hooping cough, to cold or inclement weather, for it will bring back the cough and cause inflammation of the lungs.

